# Evaluating the Impact of the COVID-19 Pandemic on Monthly Trends in Primary Care

**DOI:** 10.7759/cureus.28353

**Published:** 2022-08-24

**Authors:** Sonia Varandani, Nancy D Nagib

**Affiliations:** 1 Family Medicine, Drexel University College of Medicine, Philadelphia, USA; 2 Family Medicine, WellSpan York Hospital, York, USA

**Keywords:** cervical cancer screening, colorectal cancer screening, breast cancer screening, cholesterol control, diabetes control, hypertension control, covid-19 pandemic

## Abstract

Introduction

As the COVID-19 pandemic progressed, multiple barriers arose for patients and providers in the primary care setting. Despite the attempt to utilize telemedicine to overcome barriers, visits remained lower than pre-pandemic levels. This raises concern for preventative medicine and chronic disease management.

Methods

This study aimed to evaluate the impact of the pandemic in primary care by utilizing two years of data from a family medicine clinic. Data obtained from the electronic medical record for March 2019 through February 2020 and March 2020 through February 2021 were used to evaluate monthly trends from the year before the pandemic and the first year of the pandemic in the following six categories: hypertension control, diabetes control, lipid profile screening, breast cancer screening, colorectal cancer screening, and cervical cancer screening.

Results

The paired t-tests found a significant difference in the averages between the two years for all categories except hypertension control. The results for chi-square demonstrated a significant difference in four months for cervical cancer screening, five months for hypertension control and colorectal cancer screening, nine months for diabetes control and lipid profile screening, and 10 months for breast cancer screening.

Conclusion

These results show a profound impact of the pandemic on both preventative medicine and chronic disease management. This study had a large sample size but is not generalizable to the entire population. These results can help guide quality improvement measures going forward. However, further research is necessary to better understand the full extent of COVID-19’s impact on primary care.

## Introduction

The COVID-19 pandemic has strained the health care system, and there are many questions about the lasting impacts of this pandemic. Telemedicine was an attempt to respond to the decreased access to in-office visits at primary care practices [1]. However, it could not fully bridge the gap to return to pre-pandemic levels for multiple reasons. Some patients experienced technical difficulties while attempting to transition to telemedicine, and many expressed concerns about insurance reimbursement for virtual visits [2]. In 2020, many patients were afraid of COVID-19 exposure and canceled in-person appointments, and ambulatory practices had additional barriers [3]. Primary care practices had a shortage of personal protective equipment and lost personnel due to COVID-19 exposures [3]. All these issues culminated in a 60% drop in ambulatory visits during the early months of the pandemic [4]. This has implications for preventative medicine and chronic disease management. The decrease in cancer screenings has the potential for missed or late stage diagnoses of cancer. With 60% of Americans living with at least one chronic condition, regular monitoring and follow-up on these chronic health problems is crucial, especially for conditions such as type II diabetes mellitus [5]. Further research is needed to fully comprehend the lasting impacts. The purpose of this study is to evaluate trends in screening and chronic disease management at a family medicine center by comparing data from the year before the pandemic began to that of the first year of the pandemic.

## Materials and methods

Data were collected for this study using available electronic medical record information from the Thomas Hart Family Practice Center in York, PA. Because the first COVID-19 case in Pennsylvania was detected on March 6, 2020 [6], the data were obtained for March 2019 through February 2021. Data from March 2019 through February 2020 were considered the year before the pandemic, and data from March 2020 through February 2021 were considered the first year of the pandemic.

Individual patient information was not accessed, and there was no patient interaction or intervention performed. Data were obtained retrospectively through the EPIC population health dashboard, which automatically screened 9,923 patients from the clinic. The population health dashboard included categories for chronic diseases and cancer screening that impact a majority of the clinic’s patients. While there are many chronic diseases, the categories selected needed to have a large enough sample size within the clinic population for comparison, and therefore diseases were chosen from the patient registry. Based on the concern that the pandemic has impacted cancer screening and proper monitoring of chronic disease, the following six categories from the population dashboard were selected: hypertension control, diabetes control, lipid profile screening, breast cancer screening, colorectal cancer screening, and cervical cancer screening. These six categories were selected to evaluate both preventative medicine and chronic disease management. From the electronic medical record, information for each category was given as a monthly percentage completed, including the sample size represented by each percentage. The sample size included patients 18 years or older with indications for each category. Inclusion criteria and relevant data for each category are given in Table 1.

**Table 1:** Inclusion Criteria and Relevant Data for Each Category FIT, fecal immunochemical test; FOBT, fecal occult blood test; HPV, human papillomavirus

Category	Population Within Clinic	Calculating Monthly Percentage
Hypertension control	Patients 18 years or older with a diagnosis of hypertension	Percent of patient population that had a blood pressure reading within the past year that was under 140/90
Diabetes control	Patients 18 years or older with a diagnosis of diabetes mellitus	Percent of patient population that had a hemoglobin A1c within the past year under 8%
Lipid profile screening	Patients 18 years or older with a diagnosis of hypercholesterolemia, diabetes, hypertension, coronary artery disease, or congenital heart defects	Percent of patient population that had a lipid panel within the past year
	Male patients 35 years or older	Percent of patient population that had a lipid panel within the past five years
	Female patients 45 years or older	Percent of patient population that had a lipid panel within the past five years
Breast cancer screening	Female patients between 40 and 49 years	Percent of patient population that had a mammogram within the past two years
	Female patients between 50 and 74 years	Percent of patient population that had a mammogram within the past year
Colorectal cancer screening	Patients between 45 and 75 years	Percent of patient population that had a FIT or FOBT within the past year, FIT-DNA within the past three years, sigmoidoscopy within the past five years, CT colonography within the past five years, or colonoscopy within the past 10 years
Cervical cancer screening	Female patients between 21 and 29 years	Percent of patient population that had a pap smear within the past three years
	Female patients between 30 and 65 years	Percent of patient population that had either a pap smear within the past three years or a pap smear with HPV co-testing within the past five years

After compiling all data, the information was put into IBM SPSS Statistics for Macintosh, Version 26.0 (IBM Corp., Armonk, NY) for data analysis. For each category, a one-tailed paired t-test was performed to evaluate the difference in the mean between the two years with p < 0.05. In addition, chi-square was calculated to determine a monthly comparison between the two years for each category with p < 0.05. Finally, a scatterplot was created for each category to visualize the monthly difference between years.

## Results

**Figure 1:**
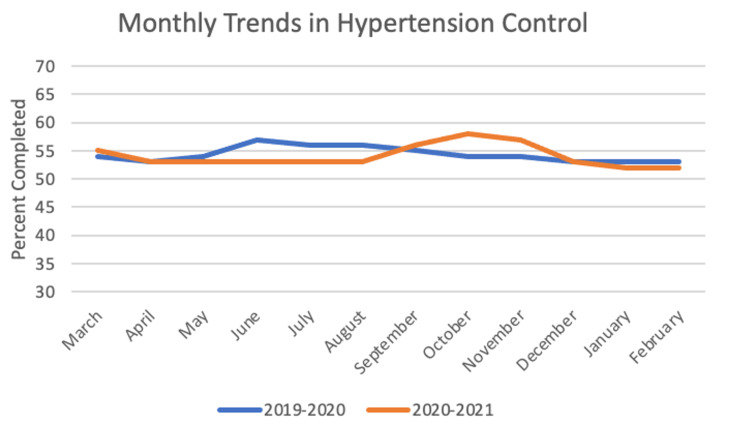
Monthly Trends in Hypertension Control

For hypertension control, the sample size was 3,352 patients. In the year before the pandemic, the frequency of completed screening was 54.33%, and for the first year of the pandemic the frequency was 54%. The paired t-test between years was insignificant with a value of 0.484 (p = 0.319). Chi-square yielded significant results with p < 0.05 for five out of 12 months: June, July, August, October, and November. These monthly trends can be seen in Figure 1.

**Figure 2:**
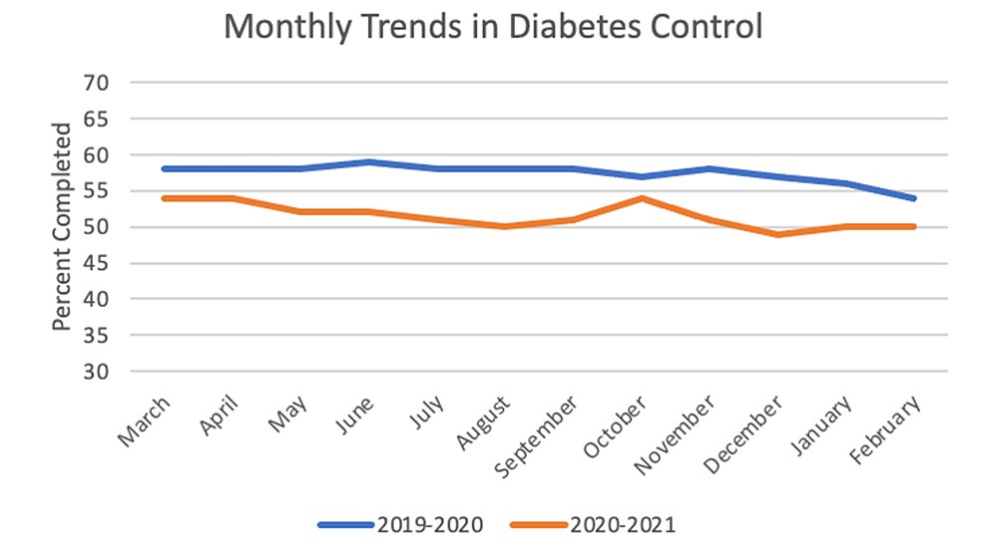
Monthly Trends in Diabetes Control

For diabetes control, the sample size was 1,269 patients. In the year before the pandemic, the frequency of completed screening was 57.42%, and for the first year of the pandemic the frequency was 51.5%. The paired t-test between years was significant with a value of 11.848 (p < 0.001). Chi-square yielded significant results with p < 0.05 for nine out of 12 months: May, June, July, August, September, November, December, January, and February. These monthly trends can be seen in Figure 2.

**Figure 3:**
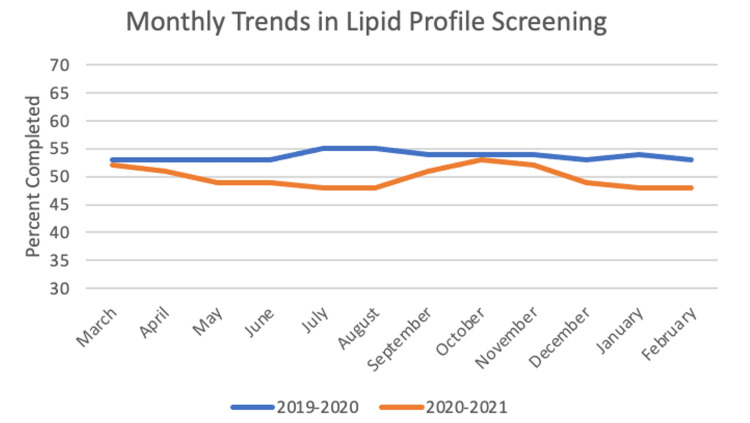
Monthly Trends in Lipid Profile Screening

For lipid profile screening, the sample size was 6,811 patients. In the year before the pandemic, the frequency of completed screening was 53.67%, and for the first year of the pandemic the frequency was 49.83%. The paired t-test between years was significant with a value of 6.249 (p < 0.001). Chi-square yielded significant results with p < 0.05 for nine out of 12 months: May, June, July, August, September, November, December, January, and February. These monthly trends can be seen in Figure 3.

**Figure 4:**
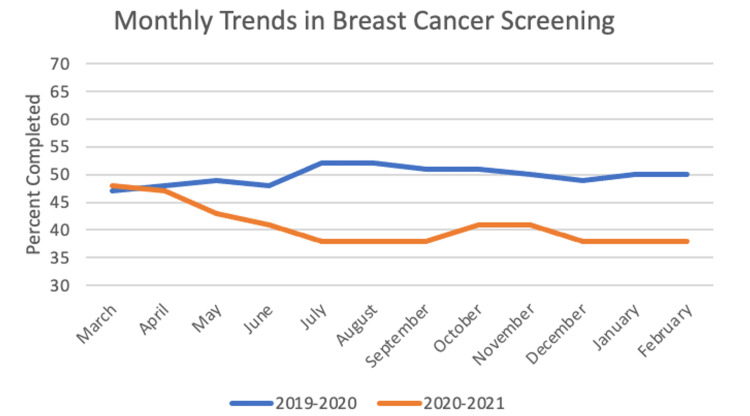
Monthly Trends in Breast Cancer Screening

For breast cancer screening, the sample size was 3,131 patients. In the year before the pandemic, the frequency of completed screening was 49.75%, and for the first year of the pandemic the frequency was 40.75%. The paired t-test between years was significant with a value of 6.34 (p < 0.001). Chi-square yielded significant results with p < 0.05 for 10 out of 12 months: May, June, July, August, September, October, November, December, January, and February. These monthly trends can be seen in Figure 4.

**Figure 5:**
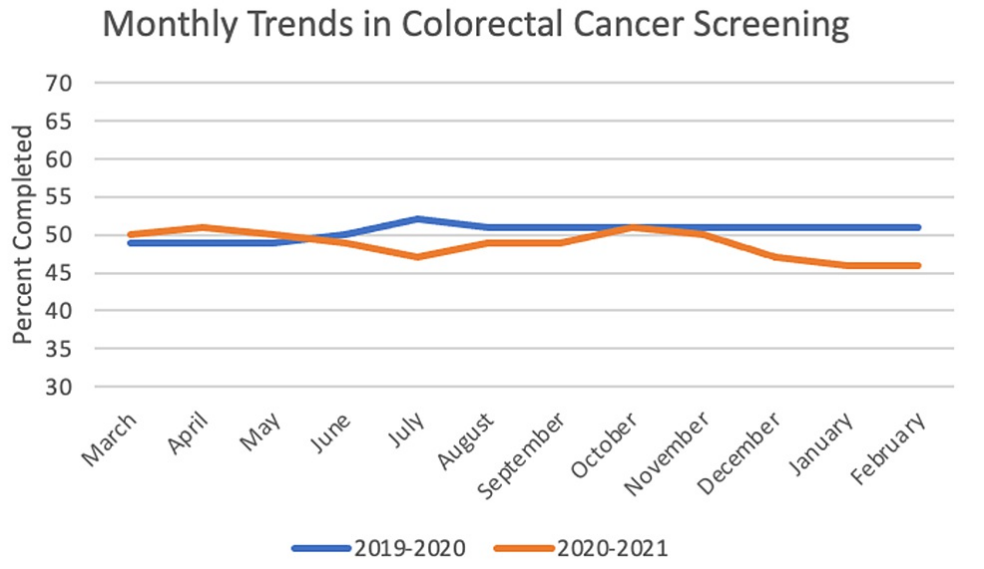
Monthly Trends in Colorectal Cancer Screening

For colorectal cancer screening, the sample size was 3,691 patients. In the year before the pandemic, the frequency of completed screening was 50.5%, and for the first year of the pandemic the frequency was 48.75%. The paired t-test between years was significant with a value of 2.399 (p = 0.018). Chi-square yielded significant results with p < 0.05 for five out of 12 months: July, August, December, January, and February. These monthly trends can be seen in Figure 5.

**Figure 6:**
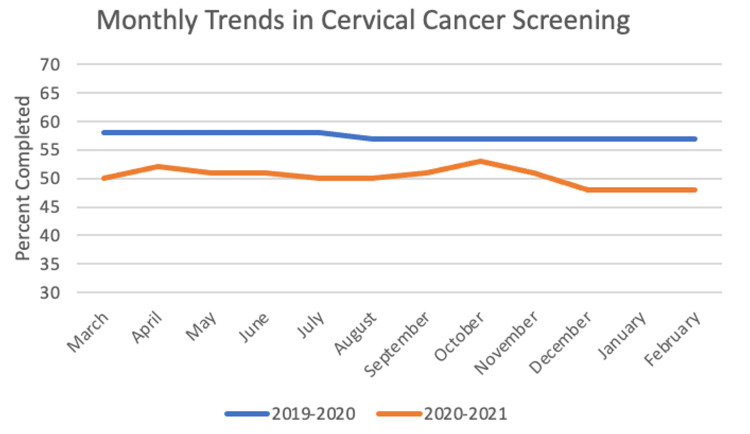
Monthly Trends in Cervical Cancer Screening

For cervical cancer screening, the sample size was 4,182 patients. In the year before the pandemic, the frequency of completed screening was 57.42%, and for the first year of the pandemic the frequency was 50.25%. The paired t-test between years was significant with a value of 16.252 (p < 0.001). Chi-square yielded significant results with p < 0.05 for four out of 12 months: May, June, July, and November. These monthly trends can be seen in Figure 6.

## Discussion

After evaluating the results from this family medicine center, it is evident that COVID-19 has been an impediment to both chronic disease management and preventative screening measures. Hypertension control appears to be the least affected by the pandemic as there was no significant difference between the averages for the two years, and only five out of 12 months had significant differences. The other five categories all showed a significant difference between averages for the two years. However, cervical cancer screening had only four months with significant differences, and colorectal cancer screening had five months. In contrast, diabetes control and lipid profile screening both had nine months with significant differences, and breast cancer screening had 10 months with significant differences.

The results of this study appear to agree with other findings in the literature. The use of hemoglobin A1c blood tests to manage diabetes decreased by 118 per 10,000 insured persons in April 2020 compared to April 2019 [7]. Between March 2020 and May 2020, primary care providers expressed concern about meeting quality of care for their patients who could not access home-based monitoring for hypertension and diabetes. In addition, the providers had difficulty ordering routine labs to monitor lipid levels and diabetes and ensuring proper referrals for diabetes [8]. Pharmaceutical dispensing data indicate concerning drops, particularly in April, May, July, and August of 2020. These decreases in dispensing medication were attributed to multifactorial barriers during the pandemic and raise concern for medication adherence for chronic diseases [9]. These studies endorse that the COVID-19 pandemic has had many impacts on chronic disease management, but the long-term impact for patients with diabetes, hypertension, hypercholesterolemia, and other conditions is not yet known.

Across the United States, facilities with cancer screening have reported deficits at 80.6% for colorectal cancer, 69.0% for cervical cancer, and 55.3% for breast cancer in comparing data from September 2019 to January of 2020 and September 2020 to January of 2021 [10]. If these delays in cancer screening continue, it is estimated that more than 80,000 Americans will experience a delayed diagnosis of cancer [11]. Based on the delayed screenings in Spring of 2020 early in the pandemic, 10,000 excess deaths are expected to be attributed to breast and colorectal cancer over the next 10 years [12]. However, the impact on preventative screening can be mitigated. Some practices were able to offset delayed screening with home tests for colorectal and cervical cancer screening [13]. Overall, these studies endorse that the COVID-19 pandemic has impacted preventative cancer screenings.

A major strength of this study is the large sample size. This family medicine center serves a large portion of the diverse patient population in York, PA, and the results provide context to the impact of the pandemic in this region. Another strength is the use of six categories of population health. These categories evaluate both chronic disease management and preventative screening, which can help understand the implications and concerns for the population going forward. However, there are limitations. This study is not generalizable to the general population as it was restricted to one center. In addition, as a retrospective study, there may be confounding variables that cannot be determined.

## Conclusions

The COVID-19 pandemic has had a profound impact on patient care in the primary care setting, and the findings from this research project can help guide future quality improvement measures, especially for breast cancer screening, diabetes control, and lipid profile screening. However, additional research is necessary to understand the impact of the pandemic on screening and chronic disease management. Future directions include evaluating outcomes for the associated conditions in this study in the upcoming years to look at the lasting impacts of the pandemic, performing this study on a larger more generalizable scale, and evaluating the impact of the same population health categories with the second year of the pandemic now complete. It is unclear how the trajectory of the pandemic will continue to influence care and how long-lasting its impact may be on preventative medicine and chronic disease management in primary care practices.
